# Gas plasma-induced bacterial PAMP release promotes skin cancer cell death

**DOI:** 10.1038/s41419-025-08283-8

**Published:** 2025-12-04

**Authors:** Julia Berner, Malin Sieben, Eric Freund, Paul Schulan, Lea Miebach, Sander Bekeschus

**Affiliations:** 1 Department of Dermatology, Venerology, and Allergology, Rostock University Medical Center, Rostock, Germany; 2 ZIK plasmatis, Leibniz Institute for Plasma Science and Technology (INP), Greifswald, Germany; 3 Department of Neurosurgery, Medical University of Vienna, Vienna, Austria

**Keywords:** Cancer microenvironment, Cancer therapy, Cell death, Proteomics

## Abstract

Infected skin tumors are challenging to treat and frequently result in tumor progression, relapses, and post-surgical complications. Moreover, bacterial infections significantly contribute to tumor therapy resistance as they release tumor microenvironment (TME)-modulating molecules. Immune or cancer cells can recognize these pathogen-associated molecular patterns (PAMPs), initiating signaling and pro- or antitumoral response. Hence, understanding PAMPs in tumor therapy may improve the understanding and efficacy of cancer treatment. Cold gas plasma treatment has shown promise in treating infected, ulcerative head and neck cancers. Here, we elucidated gas plasma-induced bacterial PAMP release and their combination with direct gas plasma exposure in skin cancer cells in vitro. Evaluating metabolic activity and viability of tumor cells revealed a significantly stronger growth-inhibitory effect of the combinatory treatment, suggesting a relevant contribution of bacterial molecules to tumor toxicity. A synergistic effect was found regarding the oxidative damage marker γH2AX that was elevated in response to the combination treatment. Cancer cells subjected to gas plasma and provoked PAMPs exhibited an altered phenotype that displayed a strikingly different chemokine and cytokine profile. Mass spectrometry analysis showed improved bacterial cell lysis by gas plasma treatment, increasing intracellular protein release of all three tested bacterial strains.

## Introduction

Skin cancer is the fourth most common cancer worldwide, leading to about 120.000 deaths annually [1]. The tumor entity comprises melanoma and non-melanoma skin cancers, whereby basal cell carcinoma represents the most common type of the latter, followed by squamous cell carcinoma. Although melanomas represent only 1% of skin cancers, they are the most aggressive form, accounting for over 80% of skin cancer deaths worldwide, and incidences are noticeably rising [2]. Despite improvements in diagnostic and therapeutic approaches, treatment remains challenging and lacks promising patient survival increases. The remarkable heterogeneity and genetic instability of skin cancer frequently result in therapy failures, evidenced by locoregional recurrences or distant metastasis [3, 4].

Furthermore, this tumor entity is the most prevalent cancer associated with bacterial infections, which can cause complicated ulcerations, exacerbating not only the health condition of afflicted patients but also their quality of life [5]. Moreover, intratumoral microbiota has been shown to affect the response to tumor-toxic treatments [6], e.g., metabolically active microbes can alter the chemical structure of anti-cancer drugs. Tumor-colonizing bacteria can also foster resistance towards treatments by modulating autophagic processes of malignant cells [7] and participate in tumor pathogenesis due to chronic inflammation, augmenting proliferation, cell transformation, angiogenesis, metastasis, and repression of antitumor immune responses [8]. Besides their potential contribution to carcinogenesis, bacterial contaminations of infected tumors can also be crucial for an effective therapy, as their inactivation can release bacterial molecules with tumor-inhibitory activity. For example, the skin commensal *Staphylococcus epidermidis* produces 6-N-hydroxyaminopurine (6-HAP) that was previously shown to reduce the growth of B16F10 melanoma cells, suggesting a protective function of these bacteria [9]. In addition, bacteria engineered to express tumor antigens elicited a strong antitumoral T-cell response, ultimately annihilating a melanoma tumor [10].

Since the immune system decisively contributes to the treatment outcome, combining immunostimulatory with conventional therapy approaches as a multidimensional strategy could potentiate an effective antitumor response [11]. Microbial products like bacterial lipopolysaccharide (LPS), DNA, or RNA generated by treatments are involved in tumor microenvironment (TME) modulation since these pathogen-associated molecular patterns (PAMPs) are recognized by pattern recognition receptors (PRR) present on the surface of immune but also cancer cells [12]. Stimulation of PRR initiates signaling pathways and inflammatory processes that can cause tumor-promoting or tumor-inhibitory effects. Besides augmented activation of immune cells like M1 macrophages [13], PAMPs can stimulate pro-apoptotic signaling in tumor cells. Through binding to Toll-like receptors (TLR), bacterial fragments can induce secretion of cytokines, chemokines, and growth factors, but simultaneously activate transcription factor IRF3-engaged caspase-dependent cancer cell apoptosis [14]. Notably, PAMPs can affect the expression of so-called immunogenic cell death (ICD) markers that can strongly prime the immune response, triggering an immunogenic tumor cell elimination [15].

To elucidate whether an alternative inactivation strategy for skin cancer-associated bacterial infections unleashes higher tumor toxicity and increased cancer cell immunogenicity due to the production of differential PAMPs, we aimed to assess the stress responses of three different skin cancer cell lines after treatment with gas plasma-generated microbial PAMPs. Gas plasma is a multi-component technology that yields antimicrobial activity by excessive deposition of diverse reactive oxygen and nitrogen species (ROS/RNS) [16]. Beneficial effects were observed in wound healing and anti-cancer therapies, rendering it a promising tool in clinical treatments [17]. Since applying oxidative stress not only inhibits bacteria but also tumor cells, thereby mediating cytotoxic and immunostimulatory effects, we also investigated the impact of a combinatorial treatment on tumor viability. Potential improvement of therapy efficacy was explored by colorimetric assays, flow cytometry, and high content imaging. To evaluate the impact of gas plasma-mediated bacterial inactivation on PAMP generation and treatment outcome, a mass spectrometry-based comparative analysis was conducted to determine the magnitude of protein release directed by gas plasma-induced oxidative stress compared to conventional heat inactivation.

## Materials and methods

### Cell culture

The human melanoma cell line A375 and the human squamous cell carcinoma cell lines A431, as well as SCC-25 (all ATCC, USA), were cultured in Roswell Park Memorial Institute (RPMI) 1640 medium (Pan Biotech, Germany) supplemented with 10% fetal bovine serum, 1% penicillin/ streptomycin, and 1% L-glutamine (all Corning, Germany). All cell lines were passaged twice weekly and incubated under standardized culture conditions (37 °C, 5% CO_2_, 95% humidity). Twenty-four hours before treatment, cells were seeded at a density of 0.5 × 10^5^ cells per well in 500 µL of fully supplemented cell culture medium in 24-well flat-bottom plates (Greiner, Austria).

### Gas plasma technology

Gas plasma treatment was conducted using the clinically approved cold atmospheric pressure plasma jet kINPen (neoplas, Germany). Argon (purity 99.9999%; Air Liquide, Germany) served as the feed gas, which was ignited with a frequency power of 1 MHz and generated power of about 1 W at a constant flow rate of 1.5 (tumor cells) or 5 standard liters per minute (bacteria). Standardized treatment heights and procedures were ensured by hovering the jet centrally over each well using a motorized and computer-controlled XYZ table (CNC, Germany). The exposure distance between the capillary of the argon plasma jet and the liquid surface was 1 cm. Following gas plasma application, evaporation was compensated by adding a pre-determined amount of double-distilled water to maintain iso-osmolarity.

### Preparation of bacterial supernatants

The bacterial strains *Staphylococcus aureus* (*S. aureus*; DSM 799/ ATCC 6538), *Staphylococcus epidermidis* (*S. epidermidis*; DSM 20044/ ATCC 14990), and *Pseudomonas aeruginosa* (*P. aeruginosa*; DSM 50071/ ATCC 10145) were grown in casein-soy-peptone-agar-bouillon (CASO; Carl Roth, Germany) at 37 °C with shaking (220 r/min). Preculture solutions were diluted with phosphate-buffered saline (PBS) to a final concentration of 10^8^ before bacterial cells were exposed to gas plasma in 24-well plates (Thermo Fisher Scientific, Germany) for different treatment times. Heat-inactivated (20 min at 65 °C in a Thermomixer Comfort; Eppendorf, Germany) and completely untreated bacterial cells served as control groups. After incubation for 1 h at room temperature, treated bacterial cells were collected and plated on Petri dishes (CASO; Carl Roth, Germany). Plates were incubated under standard culture conditions (Binder, Germany) for 24 h before colony-forming units (CFU) were counted using a semi-automated colony counter (SphereFlash; IUL, Germany) to determine the log reduction (Fig. S1a). A predefined gas plasma treatment time, ensuring a bacterial decrease of two log_10_ levels was used. For subsequent tumor cell culture experiments, supernatants were generated from untreated control (cPAMPs), heat-inactivated (hPAMPs), and gas plasma-exposed (pPAMPs) bacteria (Fig. S1b). To obtain the PAMP-enriched supernatants from bacteria being heat- or gas plasma-treated or that have remained untreated, the treated suspensions were collected 1 h after the treatment and filtered using filter syringes with a pore size of 0.45 µm. Supernatants were stored at -20°C until usage in cell culture experiments.

### PAMP-enriched bacteria supernatants in cell culture experiments

Tumor cells were either exposed to gas plasma or bacterial supernatants alone, or a combination treatment of both was performed. For incubation with PAMP-enriched supernatants, the supernatants obtained from all three bacterial strains were mixed in equal volumes (1:1:1, *v/v*) and added to the cells 24 h after cell seeding or gas plasma treatment, which was performed prior to supernatant application. For this, 250 µl of bacterial supernatant mixture or an equal volume of PBS was applied to the respective cells. Tumor cells were exposed to supernatants generated from bacteria that either have been untreated (cPAMPs), gas plasma-treated (pPAMPs), or heat-inactivated (hPAMPs). In some experiments, lipopolysaccharide (LPS; 100 ng/ml; Sigma-Aldrich, Germany) served as a positive control.

### Metabolic activity

To analyze changes in the metabolic activity of cancer cells in response to the treatment, 7-hydroxy-3H-phenoxazin-3-one-10-oxid (resazurin; Alfa Aesar, USA) was added at a final concentration of 10 µM to the cells 22 h after the treatment. Metabolically active cells convert the non-fluorescent resazurin into the fluorescent resofurin in a NADPH/H^+^-dependent reaction. Following incubation for 2 h, fluorescence was measured at λ_ex_ 535 nm and λ_em_ 590 nm utilizing a multimode plate reader (F200; Tecan, Switzerland). Resazurin without cells was used for background subtraction.

### High-content imaging

Cells were stained and analyzed using high-content imaging to assess morphological changes and determine viable and apoptotic cells following the treatments. Therefore, 500 nM Vybrant DiD cell labeling solution (Thermo Fisher Scientific, Germany) was added to the cells 46 h after treatment. Cells were incubated for 2 h before CellEvent caspase 3/7 detection reagent (1:3000; Thermo Fisher Scientific, Germany) and 1 µM DAPI (Thermo Fisher Scientific, Germany) were additionally applied to the cells. After incubation for 15 min, the plate was imaged using a high-content imaging device (Operetta CLS; PerkinElmer, Germany). Brightfield and fluorescent images were acquired with the 20× air objective. Cancer cells were fixed in the plates with 4% paraformaldehyde (PFA) for 15 min at room temperature and 24 h after treatment to evaluate the structural organization of actin filaments. This was done following washing twice with PBS, cellular permeabilization with permeabilization wash buffer (BioLegend, The Netherlands), washing, and subsequent staining with 1 µM DAPI (Thermo Fisher Scientific, Germany) and phalloidin NIR 647 Flash Red (1:1500; BioLegend, The Netherlands) overnight at 4 °C. Cells were washed twice prior to imaging (Operetta CLS; PerkinElmer, Germany). Brightfield and fluorescent images were acquired with the 40× water objective. The experiment and algorithm-based quantitative image analysis were achieved using Harmony 4.9 software (PerkinElmer, Germany).

### Flow cytometry

To analyze the expression levels of several intra- and extracellular markers, tumor cells were detached from culture plates 24 h after treatment to obtain single-cell suspensions. For surface marker staining, cells were washed and incubated with monoclonal antibodies (Table 1) targeted against (conjugate) calreticulin (CRT; PE), CD39 (PE/Dazzle594), CD40 (FITC), CD152 (BV785), CD274 (BV650), Galectin-9 (Gal-9; PerCP-Cy5.5), HLA-ABC (BV510), heat shock protein 70 (HSP70; APC) and 1 µM iFluor 840 maleimide (AAT Bioquest, USA) to distinguish live from dead cells. Following incubation for 20 min at room temperature, cells were washed and analyzed using flow cytometry (CytoFLEX LX; Beckman Coulter, Germany). To enable staining of intracellular molecules, cells were fixed (fixation buffer) and permeabilized (permeabilization wash buffer; all BioLegend, The Netherlands) following detachment. After washing, monoclonal antibodies targeted against (conjugate) γH2AX (APC/Fire750) and 10 µM 4′,6 6-diamidino-2-phenylindol (DAPI; Thermo Fisher Scientific, Germany) were added to the cells and incubated for 1 h at 4 °C. Cells were washed, and samples were acquired by flow cytometry. Quantitative analysis was performed using *Kaluza* 2.2 software (Beckman Coulter, Germany).

**Table 1:** Monoclonal antibodies used for cell surface staining.

Ligand	Fluorochrome	Clone	Supplier	Cat#
CRT	PE	FMC75	Enzo Life Science	ADI-SPA-601PE-D
CD39	PE/Dazzle594	A1	Biolegend	328224
CD40	FITC	5C3	Biolegend	334306
CD152	BV785	BNI3	Biolegend	369624
CD274	BV650	29E.2A3	Biolegend	329740
Galectin-9	PerCP/Cy5.5	9M1-3	Biolegend	348910
HLA-ABC	BV510	W6/32	Biolegend	311436
HSP70	APC	N27F3-4	Novus Biologicals	NB110-96425APC
γH2AX	APC/Fire750	2F3	Biolegend	613422

*AF* Alexa Fluor, *APC* Allophycocyanin, *BV* Brilliant Violet, *Cy* Cyanin, *FITC* fluorescein isothiocyanate, *PE* phycoerythrin, *PerCP* peridinin-chlorophyll-protein.

### Cytokine and chemokine analysis

Quantification of inflammatory mediators, such as cytokines and chemokines, was performed using a bead-based sandwich multi-analyte assay (LEGENDplex; BioLegend, The Netherlands) according to the manufacturer’s instructions. The experiment was carried out with four technical replicates for pooled supernatants of three independent biological replicates. The assay panel contained beads targeted against human interleukin (IL)1β, IL6, IL8, IL10, IL12p70, IL17A, IL18, IL23, IL33, interferon (IFN) α2, INFγ, monocyte chemoattractant protein (MCP) 1, and tumor-necrosis factor (TNF) α. Briefly, supernatants were collected from the tumor cell cultures and incubated with fluorescent detection antibodies. For detection, samples were measured using flow cytometry (CytoFLEX LX; Beckman Coulter, Germany). Absolute concentrations were calculated against standard curves using LEGENDplex 8.0 software (Vigene Tech, USA).

### Mass spectrometry sample preparation

Bacterial supernatants were lyophilized, and dried samples were reconstituted in 1 ml PBS. Proteins were precipitated in an excess of acetone (1:4, *v/v*; Carl Roth, Germany) overnight at -20 °C. Following centrifugation, the supernatant was removed, and the pellet was gradually dissolved in 500 µl PBS. An intermediate desalting step with subsequent reduction, alkylation, and tryptic digestion (ratio 1:20) was carried out using the S-trap purification and digestion protocol according to the manufacturer’s instructions (ProtiFi, USA). Briefly, reduction was performed using 30 mM dithiothreitol (DTT; Merck, Germany) at 90 °C for 10 min, and was followed by alkylation with 60 mM iodoacetamide (IAA; Merck, Germany) in the dark for 30 min at room temperature. The protein suspension was transferred to the S-Trap microcolumn, and proteins were digested with sequencing-grade trypsin (V5111; Promega, Germany) overnight at 37 °C. The digestion was stopped by acidification with formic acid, preparing the peptides for STAGE-Tip purification. In-house C-18 reversed-phase STAGE-Tips incorporating Empore SPE discs (Supelco; Merck, Germany) were used for peptide purification. Peptides were loaded onto the columns, washed thrice with 0,5% formic acid/water solution, and eluted into MS vials with 80% acetonitrile/0.5% acetic acid solution. Samples were concentrated by vacuum centrifugation at 30 °C and reconstituted in 20 µL 0,1% formic acid/water solution. Samples were stored at −80 °C until measurement by mass spectrometry.

### Liquid chromatography-tandem mass spectrometry (LC-MS/MS)

Measurements were performed on a Dionex UltiMate 3000 RSLCnano HPLC system interfaced with an Exploris 480 mass spectrometer using a nano-electrospray ion source. Analytes were isolated on a PepMap C18 trap column (5 μm particles, 20 × 0.1 mm) and separated on a PepMap C18 analytical column (3 μm particles, 150 × 0.075 mm) using a two-buffer system. Buffer A contained 0.1% formic acid in MS-grade water, and buffer B contained 0.1% formic acid in acetonitrile/water (95:5, *v/v*). Separation was conducted by a gradient of 0–85% buffer B over 65 min with a flow rate of 300 nL/min, while the column oven was set to 40 °C. Peptides were ionized by electrospray ionization (NanoSpray Flex source; Thermo Fisher Scientific, Germany) in positive ion mode ( + 2 kV) while the transfer capillary was set to 250 °C. Measurements were conducted in data-dependent acquisition (DDA) mode with a full scan range of 350–1200 m/z (resolution 120 000 at 200 m/z), a target of 5 × 10^3 ^ions, and up to 15 data-dependent MS/MS scans with higher energy collision dissociation (HCD, maximum injection time 50 ms, isolation width 1.0 m/z, NCE 30%, R = 15,000 at 200 m/z). Dynamic exclusion was set to 30 s. Three technical replicates were measured using LC-MS/MS, each containing twenty pooled biological replicates.

### Mass spectrometry data analysis

The MS raw data were extracted and analyzed with the Proteome Discoverer 2.5 software (Thermo Fisher Scientific, Germany). FASTA files for the bacterial strains were obtained from UniProt and used to match the extracted MS/MS spectra and common background contaminants. MS Amanda was used as a search engine with the following parameters: peptide mass tolerance = 10 ppm, fragment mass tolerance = 10 ppm, cleavage specificity = trypsin, missed cleavages = 2, and total common modifications = 2. Data processing and statistical analysis of quantitative proteomics were performed using Perseus (version 2.0.6.0). Protein FDR was set to 1%, and the minimum ratio count for unique peptides was set to 2. Peaks detected as “High” in at least two out of three replicates in at least one group were considered for quantification. P-values obtained from a paired *t*-test were used to determine the significance of differences in protein abundances between gas plasma-treated and heat-inactivated bacterial supernatants. P-values (adjusted) ≤0.05 were considered statistically significant. Classification of differentially expressed proteins was performed by Gene Ontology (GO) pathway analysis using the PANTHER database. Enrichment and functional network analysis were conducted and visualized using Database for Annotation, Visualization, and Integrated Discovery (DAVID), STRING, and Cytoscape. The proteome data were deposited into the PRIDE database (https://www.ebi.ac.uk/pride/) under the accession number PXD070469.

### Statistical analysis

Data graphing and statistical analysis were done using *Prism* 9.51 (GraphPad Software, United States). Data show mean ± standard error of the mean (SEM) of at least three independent biological replicates comprising three technical replicates each, if not indicated otherwise in the figure legends or method section. One-way or two-way analysis of variance (ANOVA) was used to determine significant differences between groups, if not stated otherwise in the figure legends. Levels of significance were as follows: *p ≤ 0.05, **p ≤ 0.01, or ***p ≤ 0.001. Replicates were excluded from analysis if measurement was considered as unreliable due to technical issues (e.g., sample outside detection limit, inappropriate acquisition), or identified as outlier using the *Identify outliers* function (ROUT, Q = 1%) in Prism 9.5.1.

## Results

### Combination of gas plasma technology with pPAMPs improves treatment efficacy

**Fig. 1:**
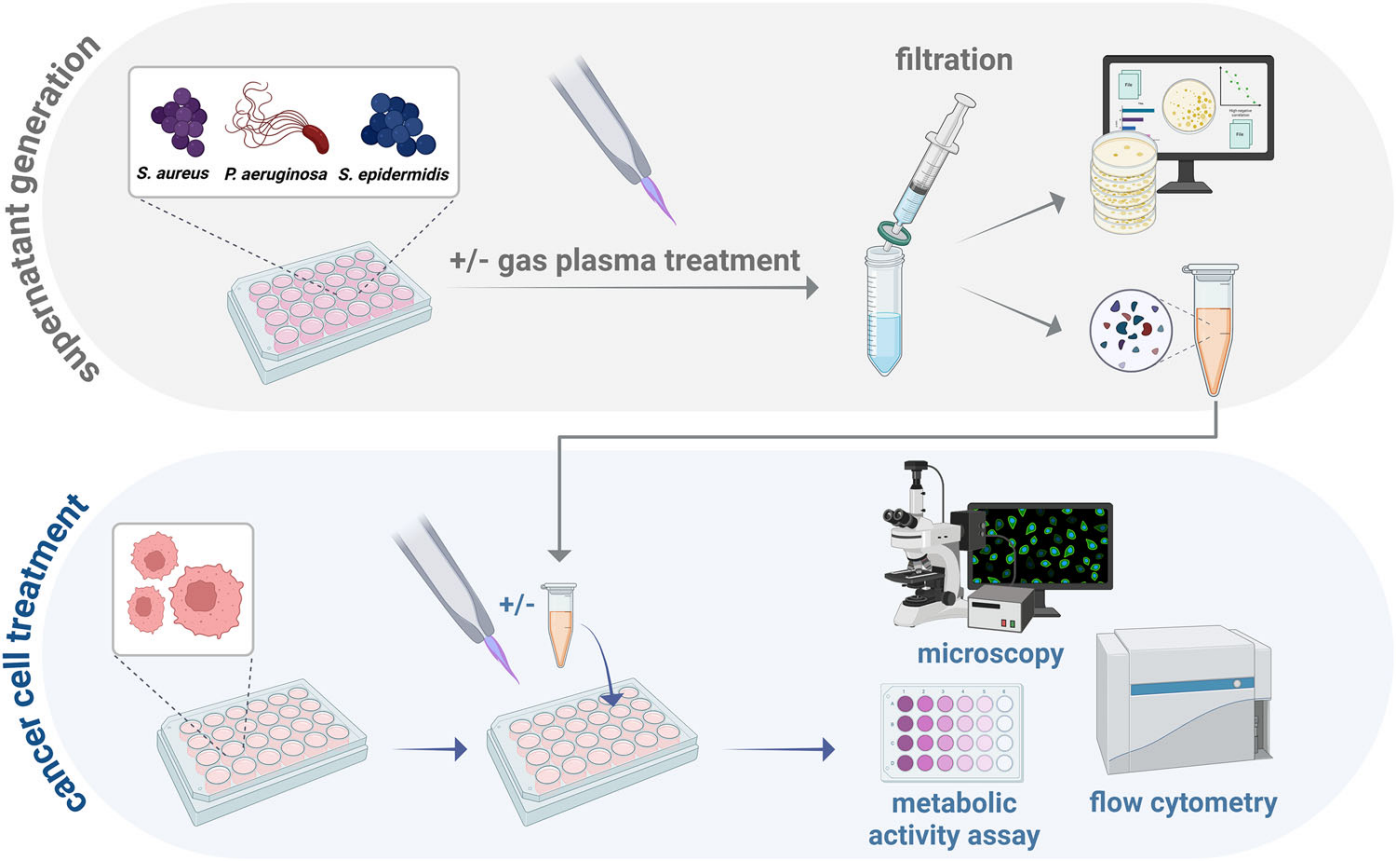
Schematic overview of the study design. Bacterial suspensions were exposed to gas plasma before supernatants containing gas plasma-induced PAMPs (pPAMPs) were collected (top) and added to untreated or gas plasma-treated tumor cells (bottom). Treatment-associated stress responses were evaluated using different downstream analyses.

**Fig. 2:**
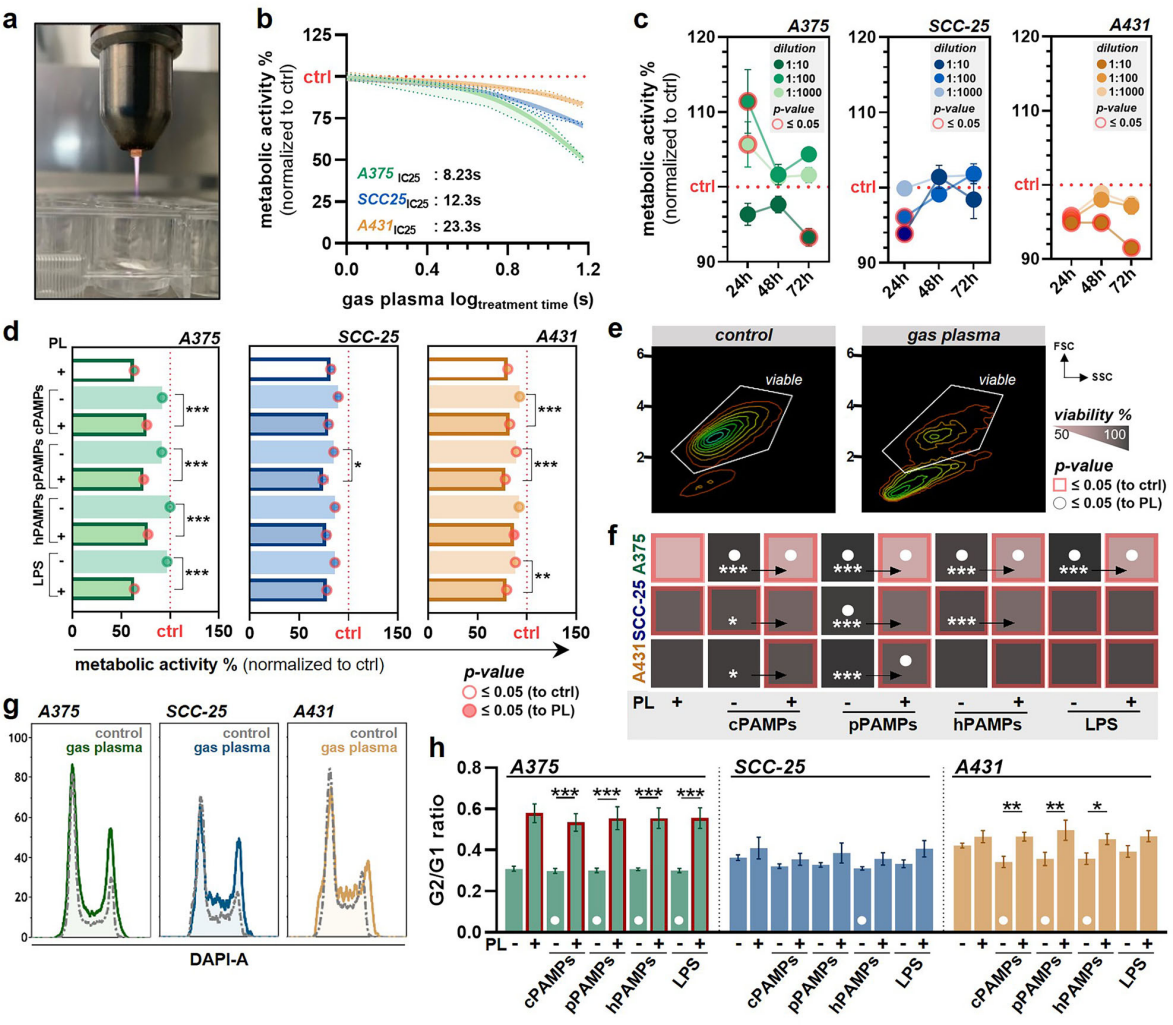
Administration of gas plasma technology in combination with pPAMPs results in significantly increased tumor inhibition. **a** representative image of gas plasma treatment; **b** metabolic activity of A375, SCC-25, and A431 cells 24 h post gas plasma treatment and calculated IC_25_ values; **c** metabolic activity of A375, SCC-25, and A431 cells 24 h, 48 h, and 72 h after treatment with three different dilutions of bacterial pPAMP-containing supernatants, statistical analysis was performed using two-way analysis of variance (ANOVA); **d** metabolic activity of A375, SCC-25 and A431 cells 24 h after gas plasma or PAMP treatment alone or combination treatment, statistical analysis was performed using one-way ANOVA; **e** representative flow cytometry contour plots of the viable cell population; **f** quantification of viable cells 24 h after gas plasma or PAMP treatment alone or combination treatment, statistical analysis was performed using two-way ANOVA; **g** representative flow cytometry histogram of DAPI intensity for cell cycle analysis; **h** G2/G1 phase ratio in A375, SCC-25 and A431 cells 24 h after gas plasma or PAMP treatment alone or combination treatment, statistical analysis was performed using one-way ANOVA. cPAMPs = PAMPs derived from untreated bacteria. ctrl = control. hPAMPs = PAMPs derived from heat-inactivated bacteria. LPS = lipopolysaccharide. PL = gas plasma. pPAMPs = PAMPs derived from gas plasma-exposed bacteria.

**Fig. 3:**
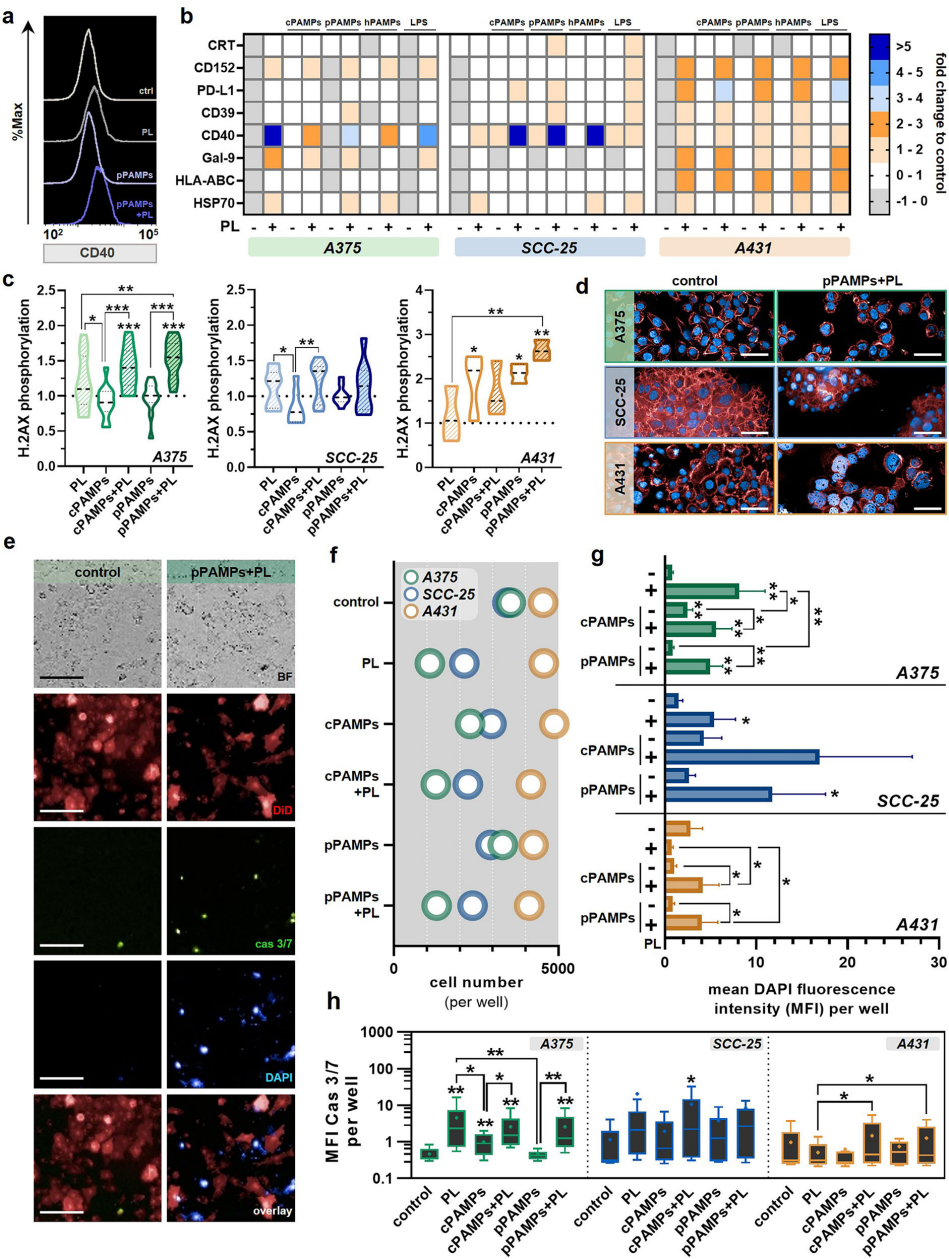
Combination treatment of gas plasma and pPAMPs significantly alters the expression of intra- and extracellular markers. **a** representative flow cytometry intensity histograms of CD40 levels in control and treated SCC-25 cells; **b** heatmap showing medians of calculated expression changes of eight different surface markers in response to gas plasma or PAMP treatment alone or a combination treatment of both (*n* = 4); **c** quantification of intracellular γH2AX expression in A375, SCC-25 and A431 cells 24 h after gas plasma or PAMP treatment alone or combination treatment, violin plots show median (indicated as stacked line) and quartiles (indicated as dotted line), statistical analysis was performed using one-way ANOVA (*n* = 4); **d** representative images of actin cytoskeleton and nucleus staining in control and treated tumor cells; **e** representative images of DiD, caspase 3/7 and DAPI staining in control and treated A375 cells; **f–h** fluorescence microscopy-based quantification of the cell number per well 24 h after gas plasma or PAMP treatment alone or combination treatment (**f**) (*n* = 4), DAPI intensity per well (**g**) (*n* = 4), and caspase 3/7 intensity per well (**h**) (*n* = 4) 24 h after gas plasma or PAMP treatment alone or combination treatment, data is shown as box and whiskers (Tukey; mean is shown as ′+′), statistical analysis was performed using unpaired *t* test (one-tailed). cas 3/7 = caspase 3/7. cPAMPs = PAMPs derived from untreated bacteria. ctrl = control. hPAMPs = PAMPs derived from heat-inactivated bacteria. LPS = lipopolysaccharide. PL = gas plasma. pPAMPs = PAMPs derived from gas plasma-exposed bacteria.

Gas plasma has exhibited antitumoral activity due to growth-inhibiting efficacy towards cancer cells and bacterial contaminations, frequently occurring at advanced stages of head and neck cancer. To investigate whether the bilateral interactions between gas plasma-induced tumor toxicity and bacterial inactivation affect treatment outcomes, we evaluated the effects of a combination treatment with gas plasma and pPAMPs on cancer cells. Therefore, three bacterial strains (*Pseudomonas aeruginosa*, *Staphylococcus aureus*, and *Staphylococcus epidermidis*) were exposed to gas plasma, and supernatants were collected 24 h after treatment. Filtrates of all strains were mixed (1:1:1, *v/v*) and used for cancer cell treatment alone or in combination with gas plasma (Fig. 1). The gas plasma inhibitory concentration 25 (IC_25_) of A375, A431, and SCC-25 cells was predefined for subsequent experiments using the resazurin assay (Fig. 2a). The melanoma cells A375 showed the highest gas plasma sensitivity (Fig. 2b). Additionally, different dilutions of pPAMP-enriched supernatants were tested for their toxicity over 72 h. Still, only the 1:10 dilutions showed a significant reduction of the metabolic activity in all tumor cell lines (Fig. 2c). To maximize the biological impact of the treatment, the following experiments were conducted with undiluted bacterial PAMPs. In SCC-25 and A431 cells, applying pPAMPs alone induced a significant reduction in metabolic activity comparable to that provoked by LPS administration. In contrast, treatment with cPAMPs showed no impact on cellular metabolism. Furthermore, the combination of gas plasma with pPAMPs strikingly augmented tumor toxicity in all cell lines, being even more substantial than gas plasma exposure alone (Fig. 2d). Flow cytometric analysis of cell viability following treatment (Fig. 2e) revealed a significantly increased efficacy by combining gas plasma with pPAMPs compared to single treatments (Fig. 2f). Correlation analysis revealed a significantly strong relation of determined viabilities with the metabolic activity (Fig. S2). By assessing the percentage of cells in different cell cycle phases, changes in G2/G1 ratios were observed in all three tumor cell lines in response to treatment (Fig. 2g). Especially cells exposed to gas plasma alone or in combination with pPAMPs showed striking differences over the untreated control and corresponding mono-treatment, indicating cell cycle arrest (Fig. 2h). In order to explore potential alterations in the expression of surface molecules, eight different extracellular markers associated with tumor immunogenicity, e.g. CD40, were quantified using flow cytometry (Fig. 3a). While nearly all targets showed markedly upregulated levels in A431 cells following gas plasma and combination treatments, only CD40 and HSP70 were found in strikingly higher abundancies on the surface of treated A375 and SCC-25 cells (Fig. 3b). Quantification of the intracellular oxidative stress marker γH2AX led to the detection of significantly higher concentrations thereof in A375 and A431 cells exposed to gas plasma in combination with pPAMPs suggesting synergistic effects. Furthermore, mono-treatments did not result in expression changes over control, except for A431 cancer cells (Fig. 3c). High-content imaging indicated visible rearrangements of the actin cytoskeleton and nucleus enlargement in treated cells (Fig. 3d) as well as remarkable changes in cancer cell apoptosis, DAPI intensity, and cell number per well (Fig. 3e). Algorithm-based calculation of the latter unveiled superior growth impairment of A375 and SCC-25 cells after gas plasma exposure alone or in combination with PAMPs. In contrast, single administration of pPAMPs or a combined treatment inhibited tumor proliferation in A431 (Fig. 3f). Similar results were derived from DAPI (Fig. 3g) and caspase 3/7 (Fig. 3h) signal intensities, suggesting higher tumor toxicity of combination approaches.

### Combined gas plasma and PAMP treatment modified tumor cytokine release patterns

**Fig. 4:**
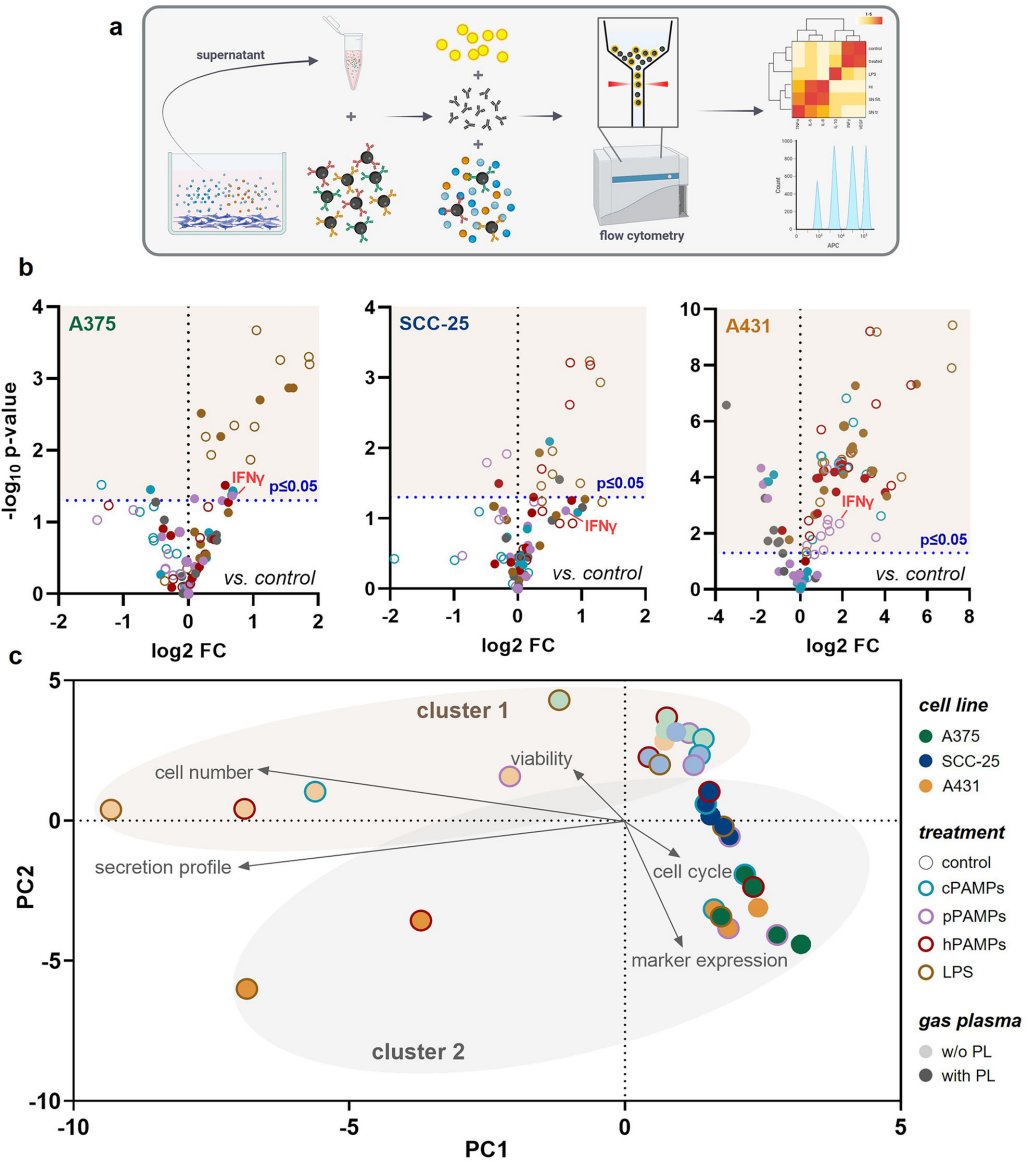
Gas plasma exposure and bacterial supernatants alter the secretion profile of A375, SCC-25, and A431 tumor cells. **a** experimental design of chemokine and cytokine analysis; **b** volcano plots show significantly altered cytokine and chemokine secretion (*p* ≤ 0.05) in response to mono- or combination treatment compared to untreated control in three different skin cancer cell lines, statistical analysis was performed using unpaired *t* test (two-tailed); **c** principal component analysis calculated from all generated data revealed two major clusters. cPAMPs = PAMPs derived from untreated bacteria. ctrl = control. hPAMPs = PAMPs derived from heat-inactivated bacteria. LPS = lipopolysaccharide. PL = gas. w/o = without. pPAMPs = PAMPs derived from gas plasma-exposed bacteria.

As inflammation has a pivotal role in the induction of antitumoral responses and the stimulation of the immune system during cancer therapies, we aimed to analyze alterations in the secretion profile of malignant cells in response to the distinct treatment modalities. Briefly, supernatants of cells subjected to mono- or combinatory treatments were collected after 24 h of incubation before beads coated with capture antibodies targeted against 13 human soluble molecules were added. Afterward, samples were incubated with biotinylated detection antibodies, forming complexes with the corresponding capture antibody. In the last step, fluorescent streptavidin-phycoerythrin was added and bound to the complexes before measurement using flow cytometry (Fig. 4a). The cytokine and chemokines levels secreted by treated skin cancer cells were compared to those of untreated cells and notable differences were determined (Table 2). Compared to A375 cells, which primarily released significantly enhanced amounts of several secretory molecules in response to LPS, SCC-25 and A431 cells additionally upregulated the cytokine and chemokine production following application of hPAMPs alone or in combination with plasma. With regard to the cPAMP and pPAMP treatment, contradictory observations were achieved in dependence on the investigated cell line. While simultaneous administration of pPAMPs and cPAMPs with gas plasma increased the concentration of immunomodulatory proteins in A375, the respective combinatory approaches resulted in a downregulated secretion pattern in A431. Nevertheless, the cytokine INFγ was found at strikingly elevated levels after pPAMP treatment over plasma exposure alone, ranking among the highest or even being the highest released molecule in this treatment group (Fig. 4b).

**Table 2:** Absolute chemokine and cytokine levels following gas plasma or PAMP treatment alone or a combined treatment.

		control	PL	cPAMPs	cPAMPs + PL	pPAMPs	pPAMPs + PL	hPAMPs	hPAMPs + PL	LPS	LPS + PL
**A375**	**Il1β**	16.12	17.64	6.64	12.51	7.29	19.09	7.33	14.25	13.42	19.54
	**IFNα2**	0.88	0.88	0.88	0.88	0.88	0.88	0.88	0.88	1.13	0.94
	**IFNγ**	1.75	2.42	1.05	2.81	1.00	2.82	1.79	2.32	6.42	5.38
	**TNFα**	1.95	1.85	1.35	2.42	1.58	1.95	1.67	2.20	3.82	3.04
	**MCP1**	7.71	7.22	6.83	7.61	7.16	8.27	8.74	8.02	16.01	10.91
	**IL6**	144.14	101.86	100.40	96.51	117.76	109.76	135.64	110.28	296.64	163.44
	**IL8**	1781.74	2433.62	1237.96	2837.97	1422.40	2905.89	2139.15	2740.99	6451.28	5210.07
	**IL10**	1.15	1.05	1.05	1.05	1.05	1.05	1.05	1.05	1.05	1.05
	**IL12p70**	0.92	0.90	0.90	0.90	0.90	0.90	0.90	0.90	0.90	0.90
	**IL17A**	0.36	0.36	0.36	0.36	0.36	0.36	0.36	0.36	0.36	0.36
	**IL18**	13.53	10.56	12.47	12.97	13.15	14.18	16.72	11.26	22.24	15.43
	**IL23**	2.39	3.08	1.90	2.97	2.09	3.45	2.62	3.55	6.37	5.15
	**IL33**	3.29	3.34	3.22	3.22	3.22	3.52	3.22	3.22	3.97	3.78
**SCC-25**	**Il1β**	4.38	9.56	3.06	8.93	2.82	5.54	8.71	7.86	12.57	9.56
	**IFNα2**	0.88	0.88	0.88	0.88	0.88	0.88	0.88	0.88	0.88	0.88
	**IFNγ**	0.64	1.05	0.66	0.71	0.64	1.18	1.44	0.77	1.63	1.04
	**TNFα**	1.78	1.82	1.54	2.53	2.13	2.06	3.16	2.08	2.63	1.40
	**MCP1**	4.29	3.79	3.79	3.83	3.09	3.66	5.58	3.99	5.57	4.07
	**IL6**	9.21	9.31	6.35	9.83	7.97	10.28	12.30	10.10	13.57	11.65
	**IL8**	610.64	592.59	486.79	618.21	542.21	566.64	682.71	622.31	809.49	619.38
	**IL10**	1.05	1.20	1.05	1.05	1.05	1.05	1.10	1.05	1.05	1.05
	**IL12p70**	0.90	0.93	0.90	0.90	0.90	0.90	0.96	0.90	0.90	0.90
	**IL17A**	0.36	0.36	0.36	0.36	0.36	0.36	0.36	0.36	0.36	0.36
	**IL18**	4.94	3.86	5.05	5.23	4.08	5.55	8.81	4.03	10.97	4.38
	**IL23**	0.92	1.35	1.09	1.02	0.99	0.99	1.53	0.73	1.88	1.18
	**IL33**	3.22	3.23	3.22	3.22	3.22	3.22	3.22	3.22	3.22	3.22
**A431**	**Il1β**	0.83	1.99	13.60	1.21	15.57	1.86	16.58	13.70	22.84	14.90
	**IFNα2**	0.89	0.88	4.05	0.88	1.83	0.88	8.81	2.76	10.93	5.44
	**IFNγ**	1.53	1.00	13.35	1.97	5.83	1.65	15.81	12.71	15.84	16.67
	**TNFα**	1.78	1.05	6.96	2.13	4.15	1.63	8.68	5.83	10.05	9.90
	**MCP1**	119.44	10.69	681.23	50.67	288.18	33.49	4511.94	219.88	17668.00	954.52
	**IL6**	2.63	1.33	9.40	0.95	7.31	0.93	31.75	10.49	375.97	119.83
	**IL8**	1540.72	599.23	6500.26	1543.59	5463.26	1138.39	6500.26	6500.26	6500.26	6500.26
	**IL10**	4.36	1.39	6.54	1.46	7.22	1.31	5.95	2.44	8.37	3.04
	**IL12p70**	0.90	0.90	1.82	0.90	1.21	0.90	1.96	1.60	2.00	1.98
	**IL17A**	0.36	0.36	0.45	0.36	0.37	0.36	0.48	0.43	0.45	0.59
	**IL18**	4.61	2.41	18.36	4.58	11.82	3.81	17.94	17.04	17.94	19.31
	**IL23**	2.74	1.19	12.86	2.53	6.88	1.65	14.38	10.90	14.71	14.40
	**IL33**	3.33	3.22	6.59	3.23	4.18	3.22	6.63	5.70	6.89	7.32

*cPAMPs* PAMPs derived from untreated bacteria. *hPAMPs* PAMPs derived from heat-inactivated bacteria. *LPS* lipopolysaccharide. *PL* gas plasma. *pPAMPs* PAMPs derived from gas plasma-exposed bacteria.

Significant differences between treatment groups independent of the respective cell line were underlined by principal component analysis, including all investigated study parameters. Two main clusters, treatment conditions without (cluster 1) and with (cluster 2) gas plasma application, were identified to separate along PC2. Alteration in cell cycle distribution and marker expression profile strongly correlated with cellular responses in tumor cells subjected to gas plasma alone or in combination (Fig. 4c).

### Gas plasma-treated bacteria released higher PAMP levels compared to heat treatment

**Fig. 5:**
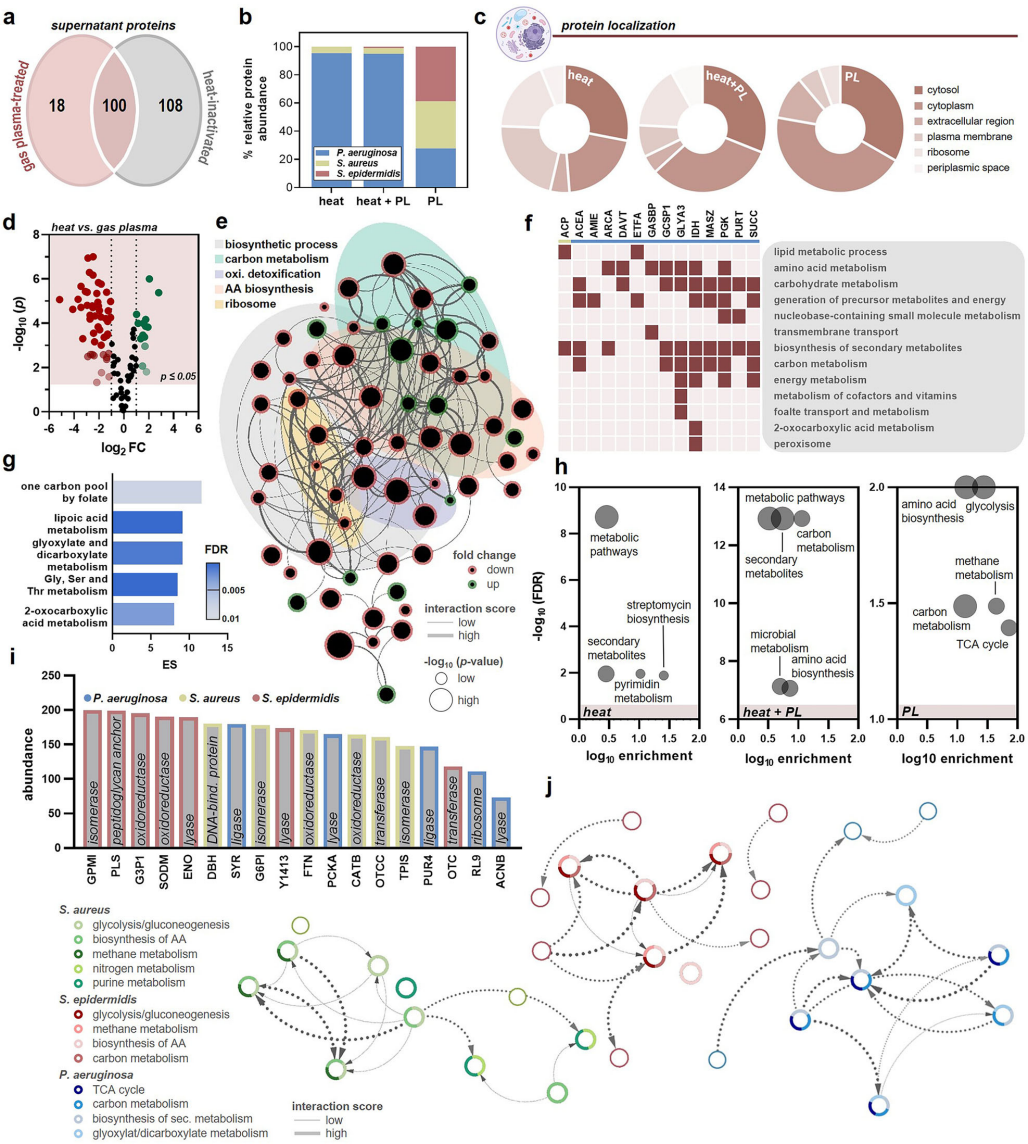
Mass spectrometric analysis of pPAMP- and hPAMP-containing bacterial supernatants. **a** Venn diagram representing the overlap between the proteins identified in pPAMP- and hPAMP-containing bacterial supernatants; **b** relative abundance of proteins from *P. aeruginosa*, *S. aureus* and *S. epidermidis*; **c** subcellular localization classification of the identified bacterial proteins; **d** volcano plot based on 100 proteins overlapping in pPAMP- and hPAMP-containing bacterial supernatants showed significant differences (*p* ≤ 0.05; fold change ≥ 2) in released proteins following gas plasma treatment over heat inactivation, statistical analysis was performed using unpaired *t* test (two-tailed); **e** PPI-network of significantly in- or decreased bacterial proteins (fold change ≥ 2) in pPAMP-containing supernatants revealed five primary cluster of function; **f** GO process classification of significantly increased proteins in pPAMP- over hPAMP-containing supernatants and (**g**) DAVID pathway enrichment analysis thereof; **h** top five enriched KEGG pathways based on proteins identified in pPAMP- and hPAMP-containing bacterial supernatants or only one of both using STRING analysis; **i, j** abundance and molecular function of 18 proteins only present in pPAMP-containing supernatants and respective interaction network. AA = amino acid. ES = enrichment score. oxi. = oxidant. sec. = secondary. TCA cycle = tricarboxylic acid cycle.

As the compositions and characteristics of the generated bacterial supernatants notably contribute to the elicited cancer cell response, cytotoxic pattern, and effects, we sought to examine the quantity and type of PAMPs released from bacterial strains following gas plasma exposure and heat inactivation. Comparative proteome analysis of gas plasma- and heat-inactivated bacterial supernatants identified 100 overlapping proteins between both groups. In comparison, 18 proteins were solely found following gas plasma treatment (Fig. 5a). Additionally, PAMP-enriched supernatants generated by the latter showed a balanced ratio (1:1:1) of proteins derived from all three bacterial strains. In contrast, heat inactivation primarily led to the production of PAMPs from *P. aeruginosa* (Fig. 5b). GO classification revealed that heat inactivation of bacteria gave rise to a strikingly higher proportion of proteins originally localized in the plasma membrane, periplasmic space, and ribosome than gas plasma exposure. Contrary, a remarkably increased amount of intracellular cytoplasmic and cytosolic proteins was released following gas plasma application, suggesting an improved bacterial cell lysis (Fig. 5c). Comparison of the abundance of proteins detected in both treatment groups underlined a differential proteome pattern following gas plasma exposure over heat inactivation since 14 and 50 molecules were produced in significantly higher or lower amount, respectively (Fig. 5d). These proteins form a tight interaction network, where biosynthetic processes, carbon metabolism, oxidant detoxification, amino acid biosynthesis and ribosome represent the five major clusters of function (Fig. 5e). Especially the 14 upregulated proteins of gas plasma-induced PAMPs mainly contribute to amino acid biosynthesis, carbon as well as carbohydrate metabolism, but also participate in pathways for energy production and biosynthesis of secondary metabolites (Fig. 5f). Functional enrichment analysis using the database for annotation, visualization, and integrated discovery (DAVID) confirmed and further specified a substantial and highly significant overrepresentation of proteins associated with carbon, lipoic acid, glyoxylate, dicarboxylate and amino acid metabolism (Fig. 5g). In general, heat inactivation resulted in release of bacterial proteins involved in metabolic pathways and biosynthesis of secondary metabolites as well as pyrimidine. By contrast, gas plasma exposure provoked the generation of a more diverse protein set that is part of the amino acid biosynthesis, glycolysis, tricarboxylic acid (TCA) cycle, carbon and methane metabolism (Fig. 5h). The top five most abundant proteins solely identified in pPAMP-containing bacterial supernatants were all assigned to the bacterial strain *S. epidermidis*, from which not a single PAMP could be found in the heat-inactivated group (Fig. 5i). The majority of the 18 gas plasma-induced proteins exert a molecular function as isomerase, oxidoreductase or lyase and show a highly interactive protein-protein-interaction network (Fig. 5j). While proteins derived from *S. aureus* and *S. epidermidis* are engaged in glycolysis/gluconeogenesis, methane metabolism and biosynthesis of amino acids, detected PAMPs linked to TCA as well as glyoxylate/dicarboxylate metabolism were exclusively designated to *P. aeruginosa*.

## Discussion

Current skin cancer treatment modalities lack effective approaches to successfully eradicate all types of malignant cells and strikingly improve treatment outcomes, e.g., 5-year survival rates. Thus, novel options to augment cancer therapy efficacies are urgently needed. Recently, bacteria-based cancer immunotherapy has attracted increasing attention as an emerging cancer control strategy. It represents an innovative oncological technique that benefits from the potential of bacteria and their metabolites to destroy tumor cells, partly by stimulating the host immune system to recognize and attack cancer cells [18]. As immune evasion is a fundamental hallmark of cancer that conduces to the tumor’s uncontrolled growth and emerges from tumor-initiated defective immune cell function, the local tumor microenvironment (TME) decisively shapes the antitumoral response [19]. Since bacteria can target and colonize a tumor’s core area, their inactivation can initiate a cascade of molecular reactions in the TME. Under cell-damaging influences, many pathogen-associated molecular patterns (PAMPs) are derived from bacteria, including lipopolysaccharides (LPS) and flagellin, and their recognition directs specific biological effects on other cells [15]. For example, PAMPs can amplify the interplay between tumors and the immune system [20, 21], e.g., by activating the migration of innate immune cells, such as macrophages, dendritic cells, and neutrophils, to the tumor, redirecting the antitumor immune response and fostering tumor cell eradication [22].

Multiple studies confirmed the antimicrobial potential of medical gas plasma application in vitro, in vivo, and in clinical trials [23–27]. To elucidate the potential therapeutic benefits of gas plasma-mediated microbial elimination and -induced PAMPs on treatment outcomes and antitumoral efficacy of the gas plasma technology, the three bacterial strains *P. aeruginosa*, *S. aureus*, and *S.epidermidis* were inactivated by gas plasma treatment, and the generated PAMP-containing supernatants were applied to skin cancer cells alone or in combination with gas plasma.

Clinical case studies impressively demonstrated the promising antitumoral properties of gas plasma treatment [28, 29]. Here, patients suffering from advanced stages of head and neck squamous cell carcinoma were subjected to gas plasma treatment thrice a week in a palliative setting, and the medical gas plasma technology not only successfully reduced the amount of bacterial load on the tumor surface but also partially reduced tumor growth. That the tumor toxic effects are not only provoked by the inactivation of tumoral contaminations has been shown in several in vitro and in vivo studies that reported significant tumor cell inhibition and reduction independent of bacterial colonization [30–34]. Following gas plasma exposure, we also observed a substantial decrease in tumor metabolic activity and viability in three skin cancer cell lines. Besides its general cytotoxic impacts, such as impairment of proliferative capacity, induction of apoptosis, and cell cycle arrest, gas plasma augmented the immunogenicity of cancer cells. By increasing the surface-bound immunogenic cell death marker levels, like calreticulin (CRT) and HSP70, the gas plasma treatment indirectly triggers the host antitumor immunity, e.g., enhanced tumor cell uptake by macrophages [35, 36]. In this study, the co-stimulating protein CD40 was significantly higher expressed on the surface of gas plasma-treated cancer cells than in untreated controls. As a cell surface molecule of the tumor necrosis factor (TNF) receptor family and binding receptor of TNFSF5 (CD40L), CD40 is mainly present on antigen-presenting cells (e.g., macrophages and dendritic cells). It plays a crucial role in their activation [37]. Nevertheless, pro-inflammatory cytokines and epigenetic modulations can elevate CD40 expression on tumor cells, promoting tumor cell apoptosis that leads to cancer cell death and additionally modulates tumor immunogenicity, for example, by upregulating the production of pro-inflammatory signal molecules like IL6, IL8, and TNFα and other immunogenic cell death (ICD) markers (e.g., CD80 and ICOS-L) [38]. This can sensitize tumor cells to immune activation and trigger co-stimulation of CD4^+^ and CD8^+^ T cells [39]. This study observed a comparable or even augmented CD40 expression in response to combination treatment with pPAMPs, suggesting an interaction between bacteria-derived PAMPs and cancer cell biology. This assumption is underlined by the significantly elevated amount of the oxidative stress marker γH2AX as well as caspase-dependent induction of apoptosis and cell death in at least two of the investigated skin cancer cell lines, emphasizing a striking contribution of pPAMPs to treatment efficacy. Asadi and Soleimani made similar observations by assessing the impact of sonication-induced *S. aureus* extracts on the proliferation of MCF-7 breast cancer cells [40]. Their findings indicated a concentration-dependent cytotoxic effect paralleled by increased cell death mediated through bacterial supernatants on the malignant cells. The cytotoxic effects induced by bacteria-derived agents, e.g., enzymes, peptides, metabolites, and bacterial toxins, have been described in several other studies [20].

Mass spectrometry analysis of generated bacterial supernatants showed that gas plasma inactivation led to the release and generation of several enzymes engaged in amino acid metabolism, indicating a potential role of the provoked PAMP profiles in improving tumor toxicity of combinatory treatments. For example, PAMPs can initiate signaling pathways that stimulate apoptosis and cell death. Cell-bound exopolysaccharides of specific *Lactobacillus* strains, as well as the secondary metabolite prodigiosin isolated from *Serratia marcescens*, were found to provoke apoptosis in carcinoma cells by activation of the Beclin-1 and GRP78 signaling axis, affecting the apoptosis-associated Bcl-2 and Bak regulation [41, 42]. Besides an augmented inhibition of cancer cell proliferation by simultaneous application of pPAMPs with gas plasma-yielded oxidative stress, analysis of secretion profiles highlighted remarkable alterations in cellular chemokine and cytokine release in response to this treatment approach. Especially the amount of IFNγ was upregulated upon pPAMP administration, usually produced by natural killer (NK) cells as well as CD4^+^ and CD8^+^ cytotoxic T lymphocytes (CTL) to orchestrate the innate and adaptive immune response [43]. For example, elevated IFNγ levels have been described to increase the immunogenicity of tumor cells, thereby triggering a tumor-directed immune response, e.g., by recruitment of lymphocytes to the TME, induction of the pro-inflammatory phenotype of macrophages, DC maturation, and differentiation of naive CD4^+^ T cells into Th1 effectors [44]. Consequently, the lack of IFNγ in the TME is supposed to be a significant mechanism of tumor immune escape and strikingly contributes to cancer treatment failure [45]. Thus, enhanced secretion of these pro-inflammatory and pro-immunogenic signaling molecules in response to the combination treatment emphasized a promising potential of pPAMPs to improve treatment efficacy by TME modulation.

In addition to immune stimulation via secretion of such immunomodulatory molecules, bacterial fragments themselves, like peptidoglycan, lipopolysaccharide (LPS), lipoteichoic acid (LTA), DNA, or RNA, can be recognized by PRRs on immune cells, e.g., DCs or macrophages, to initiate an immune response [21]. LPS, as a standard part of the outer membrane of Gram-negative bacteria, represents one of the strongest immunogenic PAMPs and has been shown to stimulate the overexpression of IL6, the activation of nuclear factor kappa B (NF-*κ*B), TLR as well as signal transducer and activator of transcription 3 (STAT3), thereby enhancing DC maturation and antitumor immunity [46]. In this study, the strong antitumoral potential of bacterial component LPS was observed by significantly inhibiting tumor cell metabolism and viability.

The great potential of bacteria-derived compounds, like LPS, for combating cancer was described first at the end of the 19th century when heat-killed streptococci were injected in sarcoma patients and caused visible tumor shrinkage [47]. Indeed, by mass spectrometric analysis, we revealed a strikingly improved lysis of Gram-positive bacteria following gas plasma application rather than heat inactivation, as an equal amount of proteins from all three bacterial strains were identified in pPAMP-containing supernatants. Additionally, a substantially higher percentage of cytoplasmic proteins was detected compared to heat-inactivated bacterial supernatants, underscoring the augmented bacterial cell lysis finding. The distinct efficacy of specific microbial inactivation treatments has been reported in several studies and is subject to the structurally different walls of Gram-positive and -negative bacteria, making it challenging to design standardized eradication methods suitable for all bacterial strains [48]. Gram-positive bacteria are generally considered less susceptible to inhibitory treatments, and similar observations were made regarding gas plasma-induced destruction [49]. However, gas plasma exhibited markedly better lysis of Gram-positive *Staphylococcus* strains than heat exposure. Compared to the latter, gas plasma is a multi-component system that comprises the generation of reactive oxygen and nitrogen species (ROS/RNS) that primarily guide antibacterial effects through biochemical reactions but also UV-radiation and electromagnetic fields, which trigger physical disruption of bacteria and further contribute to improved decontamination [49].

Besides the widely accepted PAMP peptidoglycan, bacteria-derived metabolites are also known to target several cellular processes like DNA replication, transcription, and translation, resulting in apoptosis induction, suppressed tumor growth, and impaired proliferation [42]. Thus, bacterial lithocholic acid (LCA) application notably reduced proliferation, VEGF production, aggressiveness, and metastatic potential of breast cancer cells by stimulating the antitumor immune response and mesenchymal-to-epithelial-transition [50]. Moreover, enzymes and intermediates involved in the tricarboxylic acid (TCA) cycle and amino acid metabolism can modulate cellular processes and host immune responses [51]. For example, fumarate has been shown to contribute to the immune response priming of monocytes by epigenetic regulation of cytokine expression [52]. In this study, enrichment analysis of isolated bacterial proteins from pPAMPs unveiled a substantial accumulation of enzymatic proteins engaged in such metabolic processes, e.g., amino acid, carbon, and methane metabolism, TCA cycle, and glycolysis. As the central oxidoreductase of the latter, glyceraldehyde-3-phosphate dehydrogenase 1 (G3P1) was the third most abundant protein identified in bacterial supernatants following gas plasma exposure and has been proven to act as a promising PAMP during infectious diseases. It was previously shown that intraperitoneal administration of G3P1 isolated from *Lactobacillus gasseri* could redirect immunosuppressive M2 macrophages toward the pro-inflammatory M1 phenotype [53]. Furthermore, the glycolytic metalloenzyme enolase (ENO) was among the top five enriched pPAMP proteins, and previous studies verified its binding to neutrophils, eliciting the formation of neutrophil extracellular traps (NETs) that promote immune cell-mediated elimination of microbes [54]. Due to its immunostimulating properties, bacteria-derived enolase has been considered for developing protein-based vaccines [55] and, therefore, holds the potential to ameliorate the antitumor immune response upon enhanced bacterial release. In consequence, besides direct treatment of surficial tumors like head and neck cancer or other skin neoplastic lesions, gas plasma-induced PAMPs could be used as adjuvants in cancer vaccines [56].

Overall, gas plasma delineates a powerful tool and promising treatment approach to treat easily accessible cancer entities, e.g., skin cancer, to reduce bacterial load and tumor growth through general bacterial and tumor toxicity as well as PAMP-mediated tumor cell inhibition and potential immune system activation.

## Conclusion

Medical gas plasma-generated bacterial PAMPs were investigated regarding their antitumoral potential in monotherapy and combination with gas plasma technology. Both treatment modalities strikingly diminished the growth of skin cancer cells, but their combined administration showed a markedly higher efficiency in tumor cell inhibition. This was underpinned by a synergistically increased oxidative damage marker γH2AX level and a strongly divergent cytokine profile. Gas plasma exposure led to the generation of an altered bacterial protein pattern compared to heat inactivation in generated supernatants, differing in particular by improved bacterial cell lysis following gas plasma treatment that resulted in higher liberation of cytoplasmic proteins of all three bacterial strains and the enrichment of bacterial proteins associated with amino acid metabolism, glycolysis, and the TCA cycle. Conclusively, our findings indicate that the implementation of gas plasma as an adjuvant oncological treatment option could increase the chances for curative treatment due to its general toxic impact on tumor and bacterial cells, but also its great potential to produce efficacious PAMPs, which additionally act on tumor cells and could foster immune cell infiltration. Future studies should examine the possibility of gas plasma-yielded PAMPs activating the immune system and serving as cancer vaccines.

## Supplementary information

Supplementary legends

Figure S1

Figure S2

## Data Availability

Data of this manuscript are available upon reasonable request.
