# Supplementary material for: Gas plasma-induced bacterial PAMP release promotes skin cancer cell death
Source: Cell Death Dis. 2025 Dec 4;16(1):884. doi: 10.1038/s41419-025-08283-8 (PMC12698709; doi:10.1038/s41419-025-08283-8)
Supplement: Supplementary file 1 — Supplementary legends [file 41419_2025_8283_MOESM1_ESM.docx]

**Figure S1. Gas plasma treatment resulted in an exposure time-dependent bacterial inactivation.** (**a**) representative images of plated bacterial suspensions; (**b**) determination of log_10_-reduction levels following different gas plasma treatment times.

**Figure S2. Treatment-induced changes in the metabolic activity of tumor cells correlated (Spearman) significantly and strongly with cell viability.** ctrl = control. hPAMPs = PAMPs derived from heat-inactivated bacteria. LPS = lipopolysaccharide. PL = gas plasma. pPAMPs = PAMPs derived from gas plasma-exposed bacteria. w/o = without.
