# Supplementary figures and images for: Gas plasma-induced bacterial PAMP release promotes skin cancer cell death
Source: Cell Death Dis. 2025 Dec 4;16(1):884. doi: 10.1038/s41419-025-08283-8 (PMC12698709; doi:10.1038/s41419-025-08283-8)

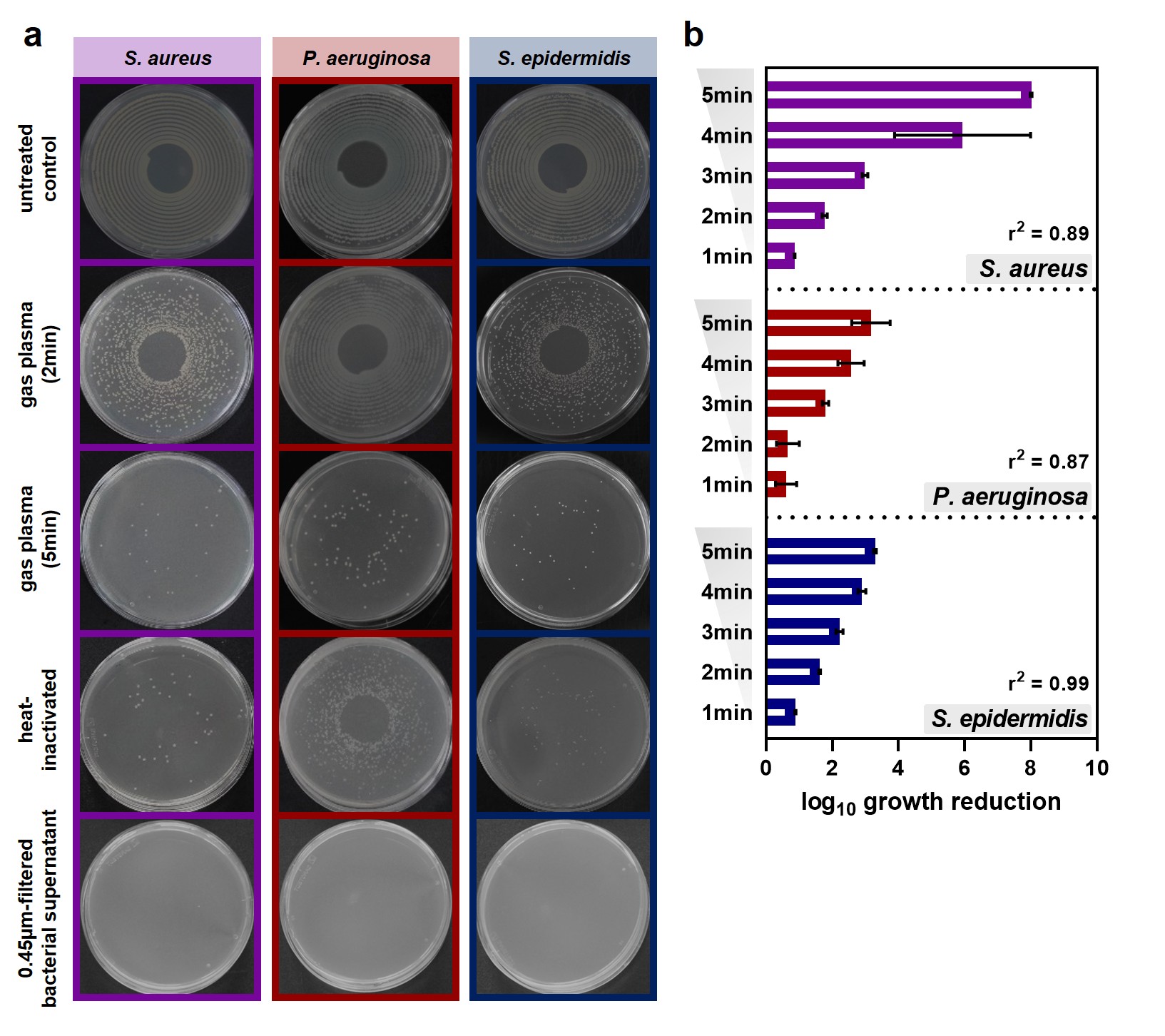

Supplement: Supplementary file 2 — Figure S1 [file 41419_2025_8283_MOESM2_ESM.jpg]

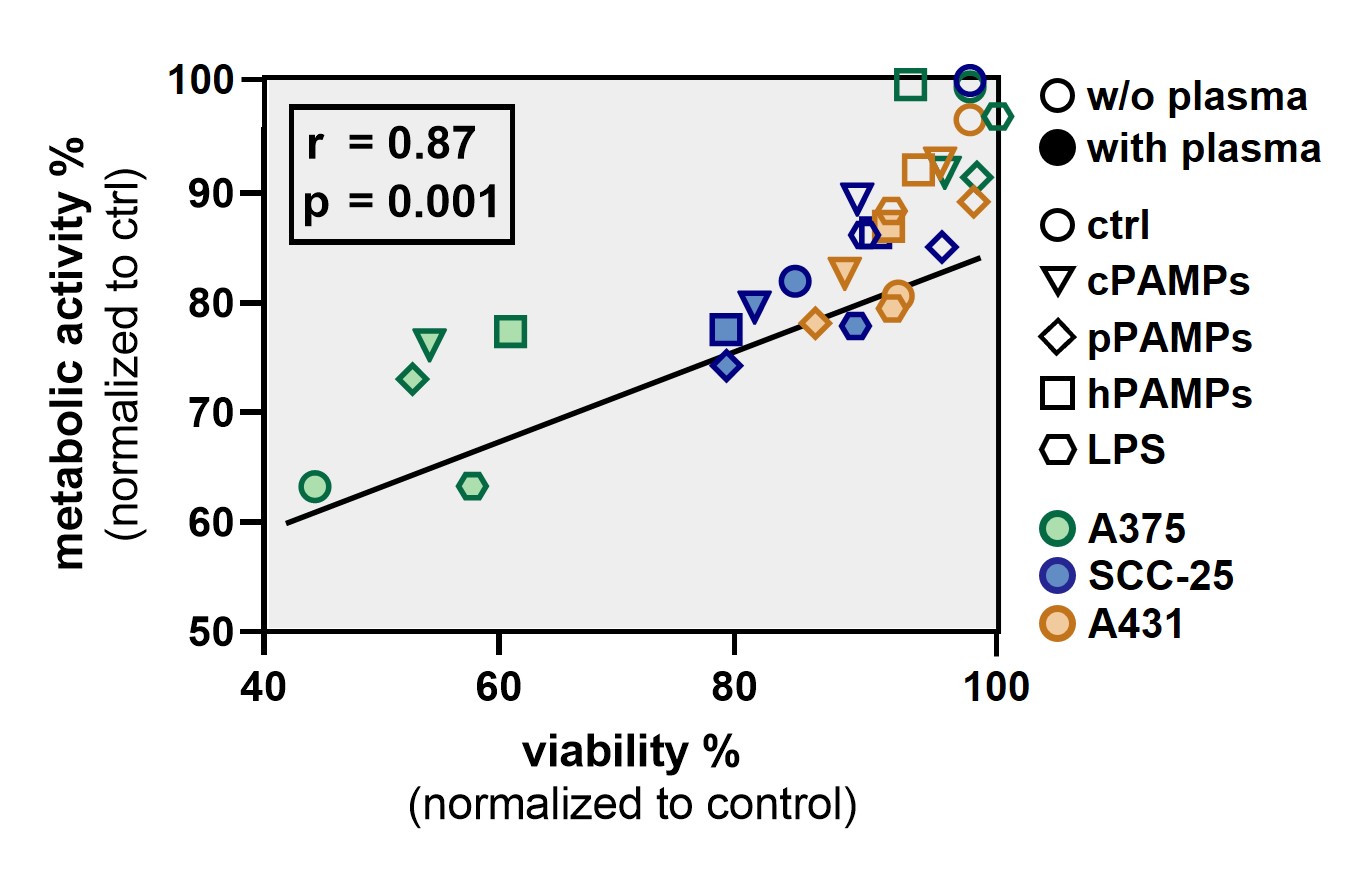

Supplement: Supplementary file 3 — Figure S2 [file 41419_2025_8283_MOESM3_ESM.jpg]
